# Suppurative Appendicitis

**Published:** 1918-02

**Authors:** I. A. Arnold

**Affiliations:** Louisville, Ky.


### SUPPURATIVE APPENDICITIS.

**By I. A. Arnold, M.D., Louisville, Ky.**

It is practically impossible to suggest any feature in connection with appendicitis which has not previously been discussed, but like other familiar subjects, while the essential points have been repeatedly outlined, there are other which have been passed without serious consideration, and it is to a few of the latter that attention will be directed in this paper.

In suppurative appendicitis all the appendicular coats are involved, and the lesion may be general or confined to some particular portion of the appendix. Pyogenic infection usually occurs on the distal side of a stricture, or in the mucosa where tissue resistance has been diminished by catarrhal inflammation, by traumatism from a foreign body, or by fecal concretions. Oftentimes the point of entry of the bacteria is exceedingly small; minute pus foci form within the mucosa, and by disintegration produce ulcers of greater or less extent; secretion is increased and becomes muco-purulent. When the peritoneal coat is implicated in the inflammatory process (as is usually true) there results a localized peritonitis with fibrinous exudate. If inflammation has not advanced sufficiently to induce peritonitis, the attack may gradually subside; but the appendix is permanently damaged and thus predisposed to subsequent inflammation, which may prove fatal in the absence of proper attention, i. e., the rational application of modern surgical principles.

In other instances pyogenic organisms penetrate the appendiceal walls, and under such circumstances the irritating effects of bacteria toxins, by agglutination of the surrounding structures, will isolate the infective focus in the appendix from the peritoneal cavity. Where bacteria penetrate the peritoneum and proliferate outside the appendix, there is produced not a diffuse peritonitis but a localized abscess. If the adhesions are dense, in rare instances such an abscess may remain small and finally disappear. More commonly, howeved, it continues to enlarge and may extend downward between the cecum and abdominal wall, or upward along the ascending colon: rupture may occur into the intestine or other viscera

The writer has observed recently two cases where the pus burrowed downward into the pelvis, also upward behind the colon, pointing in the groin and over the crest of the ilium, rendering it difficult to determine whether there was a psoas, iliac or appendicular abscess.

In some instances the diagnosis is comparatively easy, whereas in others it may be quite different. There may be no history of previous illness, but as a rule one or more attacks of catarrhal appendicitis have occurred. In a typical case of suppurative appendicitis, as in other forms, the patient is seized with sever abdominal pain accompanied by vomting. The pain may have no definite localization at first, but in a few hours becomes fixed at the right iliac fossa. After develepmont of pain there is elevation of temperature which is rarely accompanied by a chill. The rise of temperature may be slight and gradual, or may suddenly reach 104 degrees to 105 degrees F., and there is usually a corresponding acceleration of the pulse rate. At first the pulse is strong and about 100, but as septic poisoning becomes more marked it is more rapid and is temporarily increased by attacks of vomiting. The lower right quadrant of the abdominal wall is rigid, and pressure over McBurney's point elicits marked tenderness. This latter sign is probably the most important in the diagnosis of acute apepndicular lesions, but when absent and other indicative symptoms are present, the patient should be closely watched. Although the attack may terminate favorably, as a rule appendicular tension is lessened either by rupture or beginning gangrene with the extension of pus in the direction of least resistance. If nature has "walled off" the incursion in the direction of the median line—the pus burrowing into the pelvic cavity or behind the colon toward the crest of the ilium—excruiating pain does not develop until later when the more spacious locations have become filled and tension is increased. So long as the patient remain quiet in bed there is practically no elevation of temperature, but upon slight exertion it will rise to 100 degrees or 101 degrees F.

The further the abscess from the median line the more favorable the prognosis. While palpation may sometimes reveal important diagnostic symptoms, yet little may be thereby elicited to aid one in coming to a definite conclusion. Deep palpation is not only painful but exceedingly dangerous, and may cause death of the patient from rupture of an abscess which otherwise might have been successfully drained. The safer plan is to wait until the patient is anesthetized, when the exact location of the abscess can be determined. This is considered an important factor looking toward a favorable termination of the operation, of which more will be said later.

It may be well to mention some of the misleading signs obtained by palpation. When a large portion of the omentum or several coils of intestine are inflamed, edematous and "glued together" by fibrinous exudate, a mass of considerable size may be revealed if the appendix be located to the inner side of the cecum, and yet no pus be present. If the appendix be retrocecal, no mass may be descoverable excepting upon deep palpation, as it may be covered in front by the tympanitic colon, although pus may be present. If the abscess has extended upward in front or outside the colon, the mass may be detected high in the right hypochondrium, and there may be marked tenderness in the lumbar region. The right thigh will oftentimes be flexed and abducted as tension in a large abscess increases, especially in the iliac form, and there may be edema of the overlying skin and the entire right leg from pressure upon the iliac veins. Rectal and vaginal examinations are of much service in obscure cases.

Percussion over the appendicular region does not as a rule afford valuable evidence. If there be a massive exudate which extends around the appendix, dulness, flatness, or modified tympanitic resonance may be present; but in the majority of cases, unless the abscess is large and in contact with the abdominal wall, no definite information is obtainable by percussion.

Where the clinical symptoms do not enable one to determine the exact nature of the pathology present, recourse may be had to scientific measures, viz., the differential blood count. The most important phase of the blood count is a relative increase in the polynuclear cells. With little or no leucocytosis this is a positive indication of inflamamtion. The percentage of increase in polynuclear cells varies with the severity of the inflammation. If below seventy, neither pus nor gangrene is to be expected. Children have a lower percentage of polynuclear cells than adults, and in the young a percentage only slightly above seventy may indicate suppuration. Sonders says that in adults eighty-five per cent. or over of polynuclear cells always indicates a purulent exudate or a gangrenous process irrespective of the total leucocytes; ninety per cent. indicates a sever degree of infection and poor resistance; ninety-five per cent., especially with a low leucocyte count, is almost of fatal significance.

When the leucocyte count is high, similar conclusions may be drawn from the presence of a like percentage of polynuclears. The grade of general leucocytosis is a fair index to tissue reaction, and with a given percentage of polynuclears a high leucocyte count indicates a better resistance than a low one. That is to say, with a given inflammatory lesion a polynuclear percentage of eighty-five and leucocytosis of 30,000 indicate much better resistance than one of 15,000 or 20,000; with a high polynuclear coutn, if the total number of leucocytes is only 6,000 or 8,000, it indicates that the system is offering little or no resistance. According to Sonders the differential blood count and its relation to the total leucocytes is the most valuable diagnostic and prognostic aid in acute surgical diseases furnished by any method of blood examination.

The treatment of suppurative appendicitis may be classified as "condition" and operative. The term "conditional" is used because I believe there is no medicinal treatment, excepting where conditions are present which preclude operative intervention, such as Bright's disease, diabetes, certain severe heat lesions, and both extremes of age. Under such circumstances one must employ the most appropriate measures at his command without giving a favorable prognosis. The best method is to urge absolute quietude in bed, the patient being kept on the right side as much as possible so that gravitation may aid in keeping pus away from the median line. Opiates may be administered where the pain is severe and to immobilize the intestines. For local application either heat or cold (preferably ice) may be used. Vaccine may be employed, but it would probably be productive of little benefit "with a sac of pus and no drainage."

Compliance with these few general directions will about fulfill the medical indications in the treatment of suppurative appendicitis, and one may then hope for some favorable condition to arise, such as rupture of the abscess into the cecum, the colon, through the skin in the iliac region, or that the pus may become sterile and be absorbed.

In connection with operative procedures a few "Don'ts" seem appropriate, viz.:

(1) Don't get too close to the inner margin of the abscess.

(2) Don't try to remove the appendix in all cases.

(3) Don't irrigate.

(4) Don't try to drain the pus cavity with gauze.

(5) Don't keep the patient too long in bed.

An appendicular abscess should be opened as near the outer side as possible to avoid rupturing the wall which nature has constructed for protecting the peritoneal cavity. Success in this respect generally means a favorable prognosis. An extra-peritoneal opening is simply made, and the adhesions which form as the cavity closes will consist of nature's partition and the exterior wall or muscles, instead of a matting together of the intestines which would thus leave a condition favorable for the development later of the so-called "locked bowels" and other sequelae.

The abdominal incision should always be made sufficiently large to permit of careful inspection before any manipulation is attempted, and of course all pus should be evacuated. After thorough inspection, if it be found that the appendix cannot be easily removed without unduly disturbing nature's wall, it should be permitted to remain. On the other hand, if the appendix be sloughing and gangrenous, it should be removed together with all other non-adherent portions of tissue which might later act as foreign bodies; but care should be exercised not to open new channels for the extension of infection.

Irrigation is believed to be a dangerous procedure, as the fluid may be forced through a small aperture, or pressure may rupture a weak point in the plastic wall and infectious material be thus carried to other localities.

For drainage purposes a large fenestrated rubber tube is unquestionably the best. I have seen a number of operators use gauze, which not only fails to drain but occludes the opening and affords one of the most formidable nuclei for the later formation of additional abscesses.

It is believed that heretofore this class of patients have been kept too long in the recumbent position. While it would be difficult to state just what length of time they should remain in bed, as each individual case must be considered upon its merits, from six to ten days would seem quite long enough in the average case.—International Journal of Surgery.

